# Progressive Muscle Rigidity and Spasms in an Older Adult: A Diagnostic Challenge

**DOI:** 10.7759/cureus.106329

**Published:** 2026-04-02

**Authors:** Susan D Leonard, Hong-Phuc T Tran

**Affiliations:** 1 Geriatric Medicine, University of California Los Angeles David Geffen School of Medicine, Los Angeles, USA

**Keywords:** anti-gad65 antibodies, autoimmune neurologic disorder, muscle rigidity, muscle spasms, sps, stiff-person syndrome

## Abstract

Stiff-person syndrome (SPS) is a rare autoimmune neurologic disorder characterized by progressive rigidity, painful spasms, and heightened sensitivity to stimuli. We present the case of a 73-year-old woman initially diagnosed with immune-mediated myopathy attributed to statin use. Although her symptoms temporarily improved with corticosteroid therapy, she later developed severe rigidity and spasms triggered by touch and emotional stress, with preserved sensorium, findings that were inconsistent with typical myositis. Further evaluation revealed continuous motor unit activity on electromyography and the presence of high-titer anti-glutamic acid decarboxylase 65 (anti-GAD65) antibodies, supporting a diagnosis of SPS. This case highlights the importance of considering autoimmune neurologic disorders in patients with unexplained muscle stiffness, particularly when symptoms are stimulus-induced and unresponsive to standard therapies.

## Introduction

Stiff-person syndrome (SPS) is a rare autoimmune neurologic disorder characterized by progressive muscle rigidity and stimulus-sensitive spasms resulting from impaired gamma-aminobutyric acid (GABA)-mediated inhibition within the central nervous system [1,2]. With an estimated prevalence of one to two cases per million individuals, the condition is frequently underrecognized and often misdiagnosed due to overlap with inflammatory, functional, and psychiatric disorders [2,3]. Delayed diagnosis can lead to prolonged morbidity and functional decline.

Clinical suspicion is particularly challenging in older adults, where initial presentations may mimic inflammatory myopathy, including statin-associated myopathy or other medication-related muscle disease. Recognition of hallmark features, including preserved cognition, exaggerated startle responses, and stimulus-sensitive rigidity, is critical to prompt appropriate diagnostic evaluation and treatment [2]. We report a case of SPS initially attributed to statin-associated inflammatory myopathy in an elderly patient, highlighting the importance of reassessment when clinical progression does not align with the presumed diagnosis.

## Case presentation

A 73-year-old female with a history of sarcoidosis and hyperlipidemia was admitted for progressive back pain and muscle weakness. The weakness and stiffness were noted to be symmetric and predominantly involved proximal muscle groups. Initial labs showed elevated creatine kinase (CK), raising concern for inflammatory myopathy. Rheumatology suspected immune-mediated myositis, possibly triggered by statins. A complete myositis-specific antibody panel from the outside hospitalization was not available for review. Statin therapy was discontinued, and initiation of IV corticosteroids led to clinical improvement and normalization of CK. She was transitioned to oral prednisone and transferred to a skilled nursing facility for rehabilitation.

Her rehabilitation was challenged by significant deconditioning and episodic neuromuscular symptoms. Her musculoskeletal complaints included chronic back pain and nonspecific lower limb discomfort. An MRI of the lumbar spine was unremarkable, and her neurological exam showed no focal deficits. Episodes of leg cramping were also observed more frequently.

Progress with physical therapy was limited as she continued to experience muscle stiffness, painful spasms, and cramping triggered by movement and emotional stress. Her CK levels remained within the normal range. Despite a gradual steroid taper and supportive therapy, her symptoms worsened. Neurology and rheumatology initiated treatment with baclofen, diazepam, and intravenous immunoglobulin (IVIG). She demonstrated partial improvement in stiffness and spasms following treatment, although the response was incomplete. She subsequently underwent additional diagnostic evaluation, including a lumbar puncture. Cerebrospinal fluid (CSF) analysis was performed and demonstrated nonspecific findings without evidence of infection or inflammatory pathology (Table 1). Electromyography (EMG), performed at an outside institution, reportedly demonstrated continuous motor unit activity at rest in proximal and paraspinal muscles. These findings were not typical of inflammatory myopathy and instead supported a disorder of impaired central inhibitory motor control. Original EMG tracings were not available for independent review. Serum testing revealed markedly elevated antibodies against glutamic acid decarboxylase 65 (anti-GAD65) at 17,300 IU/mL. The diagnostic evaluation supporting these findings is summarized in Table 1.

**Table 1:** Summary of Diagnostic Evaluation

Investigation	Findings	Interpretation
Creatine kinase	1130 U/L (reference range: 30-200 U/L) at presentation; normalized after corticosteroid therapy.	Initial findings raised concern for inflammatory myopathy.
MRI of the lumbar spine	No structural abnormalities identified	No evidence of compressive or inflammatory spinal pathology.
Cerebrospinal fluid analysis	WBC 1 cell/µL (reference range: 0-5 cells/µL), glucose 79 mg/dL (reference range: 40-70 mg/dL), protein 46 mg/dL (reference range: 15-45 mg/dL), angiotensin-converting enzyme (ACE) 2.5 U/L (reference range: <2.5 U/L); meningitis–encephalitis panel negative; bacterial, fungal, and cryptococcal cultures negative	No evidence of infection; findings overall non-specific but compatible with noninfectious inflammatory or autoimmune process.
Electromyography (EMG): Needle EMG of proximal and paraspinal muscles	Continuous motor unit activity at rest reported in paraspinal and proximal limb muscles (outside hospital report, exact tracings unavailable)	Electromyographic findings suggestive of impaired central inhibitory motor control and supportive of stiff-person syndrome in the appropriate clinical context.
Serum anti–glutamic acid decarboxylase 65 antibodies	17,300 IU/mL (reference range: <5 IU/mL)	Supports autoimmune disorder affecting inhibitory gamma-aminobutyric acid neurotransmission and stiff-person syndrome in the appropriate clinical context.

The patient’s presentation with stimulus-induced spasms, progressive axial rigidity, and preserved sensorium raised suspicion for a neurologic disorder beyond classic inflammatory myopathy. Partial response to GABAergic agents and IVIG, in addition to EMG findings and serologic testing, supported the diagnosis of an autoimmune disorder affecting inhibitory neurotransmission, consistent with SPS.

## Discussion

This case describes a patient exhibiting hallmark features of SPS, including progressive axial rigidity, stimulus-induced spasms, preserved sensorium, positive anti-GAD65 antibodies, and electromyographic evidence of continuous motor unit activity. She did not demonstrate brainstem dysfunction or prominent autonomic instability, features more commonly seen in severe variants such as progressive encephalomyelitis with rigidity and myoclonus (PERM). CSF findings in this patient were nonspecific but did not suggest infection or alternative inflammatory pathology. This case illustrates how SPS may initially mimic inflammatory myopathy and highlights the importance of recognizing stimulus-induced spasms and preserved sensorium as key diagnostic clues. Although the initial elevation in CK and partial corticosteroid responsiveness raised concern for inflammatory myopathy, the later development of stimulus-induced spasms, preserved sensorium, and continuous motor unit activity on EMG was more consistent with SPS than with a primary myopathic process.

SPS is a rare autoimmune neurological disorder characterized by progressive axial and limb muscle rigidity and stimulus-induced spasms. First described in 1956 by Moersch and Woltman, initial case reports noted symptoms of stiffness in the neck and back with painful spasms and exaggerated startle responses [1]. The prevalence of SPS remains extremely low, estimated at one to two cases per million individuals, and the disorder is therefore frequently underrecognized and misdiagnosed as psychiatric, functional, or other neurologic conditions [2,3]. Although commonly affecting middle-aged women, SPS can present across a wide age spectrum, including pediatric and geriatric populations [4].

The pathophysiology of SPS is believed to be immune-mediated, with antibodies targeting components of GABA-mediated neurotransmission. Anti-GAD65 antibodies, which target GAD, the enzyme responsible for synthesizing GABA, are detected in approximately 60$-80% of patients with classic SPS [5]. Other antibodies, such as those against glycine receptors or dipeptidyl-peptidase-like (DPPX) protein, have been linked with severe or overlapping phenotypes, including progressive encephalomyelitis with rigidity and myoclonus (PERM). These antibodies reflect diverse immune mechanisms and reinforce the concept of SPS as a spectrum disorder. Some evidence suggests that the antibodies may interfere with vesicular GABA release or serve as disease biomarkers [6]. Additional antibodies, including anti-amphiphysin antibodies and anti-glycine receptor antibodies associated with PERM, further expand the clinical and immunologic spectrum of the disorder [7,8].

Clinically, SPS is characterized by rigidity in the axial muscles, resulting in hyperlordosis and impaired mobility. The spasms are often painful and severe and can be precipitated by stimuli such as noise, emotional stress, or light touch [8]. Autonomic features such as diaphoresis or tachycardia may be observed during attacks. Importantly, sensorium is preserved, distinguishing SPS from disorders like epilepsy or catatonia. 

SPS encompasses several clinical variants. In SPS-plus syndromes, classic features of stiffness and spasms occur alongside other autoimmune conditions such as type 1 diabetes mellitus, autoimmune thyroiditis, or pernicious anemia, supporting the broader autoimmune basis of the disorder and often correlating with the presence of anti-GAD65 antibodies [3,9]. Paraneoplastic SPS is most commonly associated with breast or lung malignancies and is typically linked to anti-amphiphysin antibodies rather than anti-GAD65 antibodies. This variant may present more acutely, and, in some cases, symptoms improve with treatment of the underlying malignancy [9]. The most severe form, progressive encephalomyelitis with rigidity and myoclonus (PERM), involves brainstem and spinal cord dysfunction, hyperekplexia, autonomic instability, and rapid progression. This variant is frequently associated with anti-glycine receptor antibodies and may result in significant disability or death without prompt immunotherapy [10]. Recognizing these subtypes is essential, as they affect prognosis, immunological evaluation, and therapeutic decision-making.

The diagnosis of SPS relies on a combination of clinical evaluation, serological testing, and electrophysiologic studies. Key clinical features include stiffness predominantly affecting the trunk and proximal limbs, exaggerated startle response, and muscle spasms provoked by sensory stimuli or emotional stress. These symptoms develop gradually but can lead to significant functional impairment [2,5].

Serologic testing focuses primarily on high-titer anti-GAD65 antibodies; however, seronegativity does not exclude the diagnosis, particularly in variants such as PERM or paraneoplastic SPS, which may be associated with antibodies such as anti-amphiphysin or anti-glycine receptor [9,10].

EMG can support the diagnosis by demonstrating continuous motor unit activity at rest, especially in paraspinal and proximal limb muscles. This persistent activity is typically suppressed with benzodiazepine administration, further supporting GABAergic dysfunction as a mechanism [5]. MRI and routine labs are typically unremarkable but are essential in excluding structural or inflammatory mimics. CSF analysis may reveal oligoclonal bands or intrathecal synthesis of anti-GAD antibodies, especially in CNS-predominant phenotypes [11]. CSF anti-GAD65 antibody testing was not available in this case because the patient’s initial diagnostic evaluation occurred at an outside institution.

Management of SPS requires a multimodal approach that targets both symptomatic relief and the underlying autoimmune process. Symptomatic therapy focuses on enhancing GABAergic transmission. Benzodiazepines, including diazepam and clonazepam, remain first-line treatment and are effective in reducing stiffness and the frequency of spasms. Baclofen, a GABA-B receptor agonist, is also commonly used and may be administered orally or intrathecally in severe cases [11].

Immunomodulatory therapy is essential, particularly in patients with high autoantibody titers or progressive disease. IVIG has demonstrated efficacy in randomized controlled trials and is considered a standard treatment for moderate to severe SPS [11]. In this case, partial clinical improvement following IVIG and GABAergic therapy was consistent with the expected treatment response in SPS. Plasmapheresis and rituximab, a monoclonal anti-CD20 antibody, have been used in refractory cases and in patients with concomitant autoimmune conditions [12]. Emerging therapies, including tocilizumab and other biologic agents, are being investigated in seronegative or treatment-resistant SPS [10].

Multidisciplinary rehabilitation is an important component for long-term management. Physical and occupational therapy can help preserve flexibility, prevent contractures, and reduce fall risk. Psychological counseling and pharmacologic treatment of comorbid anxiety and depression are often necessary, as many patients develop significant fear of movement and phobic avoidance behaviors related to painful spasms [3,5].

The clinical course of SPS is variable. Some patients achieve relative stability with appropriate treatment, while others experience progressive disability that may require assistive devices or full-time care. Falls, respiratory compromise, and reduced mobility are common complications that significantly affect quality of life [3]. Recognizing SPS as a spectrum disorder, including atypical and paraneoplastic variants, and ensuring early diagnosis with timely immunotherapy can improve outcomes and reduce the risk of severe complications. In interpreting this case, several limitations should be acknowledged, including incomplete outside hospital records, unavailable original EMG tracings, incomplete ancillary serologic testing, and limited longitudinal follow-up after transition to skilled nursing facility care.

Novel therapies such as neonatal Fc receptor (FcRn) blockers and cell-based immunotherapies are currently being explored and may have therapeutic roles in refractory cases [6]. Early recognition and treatment with immunomodulatory therapy may improve functional outcomes and reduce long-term disability [6,11]. Ongoing research and awareness are needed to promote earlier diagnosis and treatment of this disabling but potentially treatable disorder.

## Conclusions

This case highlights the diagnostic challenges in evaluating patients with new-onset rigidity and spasms. While inflammatory myopathy was initially suspected, the development of stimulus-sensitive spasms, axial rigidity, and preserved sensorium ultimately suggested a rare autoimmune neurologic disorder. Recognition of these clinical clues, together with confirmatory electromyography and antibody testing, allowed for timely diagnosis and initiation of immunomodulatory therapy. Clinicians should consider SPS in patients with progressive rigidity, particularly when symptoms are stimulus-sensitive and conventional therapies appear ineffective.
